# Comparison of the integrated organ/systems-based curriculum with the traditional subjects-based medical curriculum: Short communication

**DOI:** 10.1016/j.amsu.2021.103116

**Published:** 2021-11-25

**Authors:** Clinton Onyebuchi Obi, Moses Onosogbe, Asemota Gift Ehimen, Omotoye Olamide, Titilola Victor Toluwalase, Ovbiagele Esther, Dawodu Oluwananumi Joshua, Abdullahi Tunde Aborode

**Affiliations:** a Department of Medicine and Surgery, Ambrose Ali University, Ekpoma, Nigeria; b Department of Medicine and Surgery, Lagos State University, Lagos, Nigeria; c Oli Health Magazine Organization, Kigali, Rwanda

**Keywords:** Medical education, Reform, Curriculum, Nigeria

## Abstract

Medical education has undergone numerous reforms within the last century. Such reforms have produced numerous models of curricula across the world, which includes but is not limited to organ systems-based, problem based and traditional discipline based medical curricula. The system based of medical education in Nigeria need reform as different system are subjective to different effects on students performance and mental health. This article discuss two curricular models and identify their relative strengths and weaknesses; especially in a country like Nigeria.

## 1 Introduction

Much of the traditional discipline-based approach is based on principles advocated by Flexner and Osler in their 1910 report [1], which has produced generations of well grounded and clinically skilled physicians. The most prominent model for integration is the integration ladder introduced by Ronald Harden et al. [1]. This article discuss two curricular models and identify their relative strengths and weaknesses; especially in a country like Nigeria.

## 2 Traditional subject-based curriculum

Criticisms against the traditional subject-based curriculum include the fact that students fail to appreciate the relevance of what they learn in the basic sciences, thus lowering their motivation. The fact that much of what is presented in preclinical courses is irrelevant to what the doctor really needs to know and use in practice and lack of clinical integration to show the relationship between what they are learning now and what they would need to know in actual medical practice only exacerbates this dissatisfaction [3]. Lectures incorporated by this model are less efficient in terms of learning. Since a lecture is passive knowledge acquisition rather than active learning, it does not train a student in critical thinking or problem-solving [2].

## 3 Integrated system/organ-based curriculum

Shoemaker et al. [3] defined an integrated curriculum as “education that is organized in such a way that it cuts across subject-matter lines, bringing together various aspects of the curriculum into meaningful association to focus upon broad areas of study”. It views learning and teaching in a holistic way and reflects the real world, which is interactive [4].

Around the world, in places like the US and Canada, the Flexnerian curriculum has disappeared to permit integration between basic sciences and clinical sciences, which are taught throughout the curriculum. But this has not been the case in countries with limited resources like Nigeria.

An integrated curriculum purposefully draws together knowledge, skills, attitudes and values from different subject areas to develop a more powerful understanding of key ideas. Curriculum integration occurs when components of the curriculum are connected and related in meaningful ways by both the students and teachers.

There are two types of integration, the horizontal integration and vertical integration. Horizontal integration brings together the disciplines, topics, and subject. It refers to the provision of learning within the structure where individual departments/subject areas contribute to the development and delivery of learning a meaningful, holistic manner. By this process and links are made between the different subject areas and that learning is enriched by the connections and interrelationships being made explicit [5]. Vertical integration brings together the basic and clinical sciences.

For example, students can better grasp the complexities of physiology and anatomy if they are taught concurrently or in close proximity to each other. Think of learning the physiology of absorption and microanatomy of the GI tract together. It makes for better understanding. Thus, students in the pre-clinical years learn each organ system, moving from one organ to the next over a two-year time span.

While studying a particular organ system, a student is tasked to learn all the basic science and clinical science of that system. The systems-based model is more student-centric and provides good-quality education. There are some important areas of concern according to the report.

Innovative Curriculum Prepares Medical Students for a Lifetime of Learning and Patient Care:

• “The sequencing may prevent good correlative learning. Many diseases are multi-system multi-organ. Diabetes is an easy prototype. If a clinical lecturer discusses diabetic renal disease, and a student hasn't yet studied the renal system, learning is more superficial and mostly rote memorization, rather than deeper understanding.
• Sequencing is especially a problem in teaching pharmacology. Suppose that ACE inhibitors and beta blockers are introduced in the renal system as part of the treatment of hypertension. If a student has not studied the physiology of the heart/cardiology system yet, he/she will not comprehend the concepts of preload or after-load as they might apply.
• This model still does not address the needs of critical thinking or active learning by moving away from a lecture-based (passive knowledge acquisition) system [2].
• Integrating a curriculum is, without doubt, a complex process.” [6].

## 4 Applications of an integrated curriculum in Nigeria

According to the Nigeria Undergraduate Medical and Dental Curriculum Template*,* the aim of university education is to help students acquire the ability to think independently and to generate ideas and develop themselves; that is, they must learn how to learn that which they need to function effectively in their expected role in society [7].

Recognizing its importance, the template suggested the adoption of the integrated method of instruction. It recommended a combining of the basic medical sciences under a single integrated Core Basic Sciences.

It is worth noting the fact that almost all medical schools in Nigeria already have some form of horizontal integration. For example, a lot of students believe that pharmacology and pathology are clinical courses due to the fact that ward rounds begin around the time they start studying these courses in 4th year. But in actuality, they are still basic medical science courses in the applied sense and so attending clinical postings while studying these courses is a form of horizontal integration.

Numerous clinical applications are also presented to students in Anatomy, Physiology, and Biochemistry textbooks as well as during lectures. There is even some amount of vertical overlap between the basic science disciplines. A cardiovascular physiology lecture may start with a brief overview of the anatomy of the heart for instance. But true horizontal and vertical integration should be much more than this.

Although application of an integrated curriculum is fraught with many challenges especially in a country with limited resources like Nigeria, it can be easier for newer institutions as seen in the case of the Ziauddin Medical University (ZMU) at Pakistan unlike established medical colleges that may need a major policy reform [8].

While it may be harder, this does not mean that older medical colleges cannot implement the same principles in applying integration. Still in the case of Ziauddin Medical University, to achieve this, it was decided to have one functional Basic Health Sciences department (BHS) with departmental head. Thus, they had Faculty members with postgraduate qualifications in Anatomy, Physiology and Biochemistry but for academic purposes they function as one department [5].

In a similar fashion, the Nigeria Undergraduate Medical and Dental Curriculum Template advised that medical education units or departments be established to guide the integration process to ensure effective implementation of the curriculum and each unit or department should be staffed by medical education and content experts who will guide the negotiation between departments. These experts will also be appointed as the “lead faculty” or “course directors” for the new integrated units. The medical education unit will also be charged with monitoring curriculum implementation and ensuring regular reviews and revisions of the curriculum. The problem-based learning (PBL) approach consisting of small-group teaching sessions/tutorials was also encouraged [7].

In the case of the PBL curriculum, however, the allegedly high demands of logistics, manpower and money for its proper execution, is also considered a major issue by many. But several solutions to allay this fear can be utilized in its implementation [9].

Students in general, usually have good perceptions of the integrated medical curriculum and even more complex ones like PBL. For example, in a study at Rajeev Gandhi Institute of Medical Sciences, Srikakulam, Andhra Pradesh, India consisting of one hundred MBBS students, 35.4% of students did indicated a negative perception about it. But overall, 95.6% of students felt that integrated teaching is advantageous. In another study carried out in the Anatomy department of Lagos State University College of Medicine (LASUCOM), Ikeja, Lagos, Nigeria among 76 third year undergraduate medical students of the college, the medical students demonstrated as well as believed that, PBL was effective and more beneficial than a traditional teaching regimen as an anatomy teaching method.

The article even went on to suggest that PBL could have a role in medical colleges even in our environment [10,11], especially as a method to explore the non-cognitive domains of learning [10]. While it does suggest that an intelligent combination of using both the traditional and PBL approaches for teaching anatomy may provide the most effective training for undergraduate medical students, a simple vertical integration of the basic science disciplines in areas where needed might still be enough given the gross lack of resources in the country.

## 5 Conclusion

While the traditional method of teaching has produced regular streams of highly educated and competent doctors for decades, integration is of much importance in helping Nigerian medical schools meet global standards. A curriculum reform of this scale could be very difficult and challenging, but carefully considering and solving the issues that may arise while implementing it would be far better than simply doing nothing. As much as such a reform is needed in the earliest time possible, time should be taken to carefully gather information while attempting to implement the integrated curricula so that other universities can learn from the process and revise it suit their needs. Rather than considering only shifting to an integrated curriculum, a way of using a mix of other forms of learning like Problem-Based Learning (PBL) should also be considered, but where resources may not permit this, a simple horizontal and vertical integration might suffice.

## Declaration of competing interest

All authors declare no conflicts of interest.
